# Antitumor activities of Juemingzi (*Cassia tora* L.) on Balb/c sarcoma 180-injected mice

**DOI:** 10.3892/ol.2013.1647

**Published:** 2013-10-29

**Authors:** SHAOCHENG CHEN, GUIJIE LI, KAI ZHU, PENG SUN, RUI WANG, XIN ZHAO

**Affiliations:** Department of Biological and Chemical Engineering, Chongqing University of Education, Nan’an, Chongqing 400067, P.R. China

**Keywords:** Juemingzi, tumor, sarcoma 180, Balbc/c mice

## Abstract

The antitumor activity of Juemingzi (*Cassia tora* L.) was investigated in mice that were fed various concentrations of the compound. Although mice fed a low concentration (50 mg/kg b.w.) of Juemingzi exhibited a high tumor weight, the higher feeding concentrations (100 and 200 mg/kg b.w.) were associated with lower weight tumors. The growth rate of mouse splenocytes that were treated with 200 mg/kg b.w. Juemingzi was determined using a 3-(4,5-dimethyl-2-thiazolyl)-2,5-diphenyltetrazolium bromide assay. This rate of proliferation was higher than that achieved with 100 and 50 mg/kg b.w. Juemingzi treatment by fetal bovine serum, lipopolysaccharide or concanavalin A. Compared with the lower concentrations of Juemingzi treatment, 200 mg/kg b.w. Juemingzi significantly (P<0.05) reduced aspartate aminotransferase, alanine transaminase and blood urea nitrogen levels. A high concentration of Juemingzi (200 mg/kg b.w.) significantly (P<0.05) increased the levels of tumor necrosis factor-α and interleukin-1β cytokines compared with those of the mice that were treated with 100 and 50 mg/kg b.w. Juemingzi.

## Introduction

Juemingzi is the seed of the *Cassia tora* L. (Leguminosae) plant and has been used as a laxative and a tonic, as well as being a popular health drink (1). Pharmaceutical research has concentrated on the beneficial activities of Juemingzi, including its anti-aging, anticancer and antioxidant effects (2–5). Juemingzi contains anthraquinones, naphtho-pyrones, fatty acids, amino acids and inorganic elements (6). Types of Juemingzi with a high anthraquinone content, including chrysophanol, physcion and obtusin, may aid in cancer prevention (7).

Metastasis is a multistep process that begins when a primary tumor acquires mutations and becomes invasive. The tumor cells eventually enter into the blood or lymph (8). Metastases arise most commonly in the lung, liver, brain and bone. Notably, the lung is the most common site for systemic sarcoma metastases due to the substantial vasculature that feeds into this organ, in addition to particular trophic factors (9).

The sarcoma 180 mouse cell line is derived from a sarcoma that was carried in Swiss Webster mice and has been described to grow in multiple inbred mouse strains due to β2-microglobulin deficiency, major histocompatibility complex (MHC) class I destabilization and a lack of recognition by host cytotoxic T lymphocytes. An injection of these cells into mice results in mortality due to the accumulation of ascites fluid (10). BALB/c mice are distributed globally and are among the most widely used inbred strains that are used for animal experimentation. Balbc/c mice are often used for *in vivo* cancer research. The sarcoma 180 tumor-bearing mouse model was a staple research animal model that was used for the tumor and metastasis study (11).

The present study investigated the antitumor effect of Juemingzi in sarcoma 180-transplanted mice using a mouse model. The effects of Juemingzi at different concentrations were determined. Additionally, the serum levels and splenocyte cell proliferation were assessed.

## Materials and methods

### Preparations of Juemingzi (Cassia tora L)

Juemingzi was purchased from Yunnan Baiyao Group Co. Ltd. (Kunming, China), stored at −80°C and freeze-dried to produce a powder. A 20-fold volume of methanol was added to the powdered sample and extracted twice by stirring overnight. The methanol extract was evaporated using a rotary evaporator (N-1100; Eywla, Tokyo, Japan), concentrated and dissolved in dimethylsulfoxide (Amresco, Solon, OH, USA) to adjust to the stock concentration (20%, w/v).

### Animals

Female six-week-old Balb/c mice (n=50) were purchased from Chongqing Medical University (Chongqing, China). The mice were maintained in a temperature controlled (25±2°C; relative humidity, 50±5%) facility with a 12-h light/dark cycle and free access to a standard mouse diet and water. This study followed a protocol approved by the Animal Ethics Committee of Chongqing Medical University (Chongqing, China).

### Cell preparation

Mouse sarcoma 180 cells were purchased from the Shanghai Institute of Biochemistry and Cell Biology (Shanghai, China). The sarcoma 180 cell line was cultured for 7–10 days in the abdominal cavity of a Balbc/c mouse and the cultured cells were harvested with the peritoneal fluid and centrifuged at 300 × g for 10 min in phosphate-buffered saline (PBS). The separated sarcoma cells were suspended in PBS, centrifuged at 1,800 × g for 5 min and their concentration was adjusted to 1.0×10^6^ cells/ml by diluting in Dulbecco’s modified Eagle’s medium.

### In vivo antitumor activity assay

The sarcoma 180 cells (0.2 ml; concentration, 1.0×10^6^ cells/ml) were implanted subcutaneously in the left groin of the mice in the control and sample groups (11). The mice from the normal and control groups were fed with a normal diet and water. The sample group mice were administered 50, 100 or 200 mg/kg b.w. intragastric Juemingzi for 28 days. The mice were sacrificed using CO_2_. The tumors were then removed and weighed. The tumor growth inhibition ratio (I.R.) was calculated using the following formula: I.R. (%) = (Cw−Tw)/Cw × 100; where Cw and Tw represent the average tumor weight of the control and experimental groups, respectively.

### Serum aspartate aminotransferase (AST), alanine transaminase (ALT) and blood urea nitrogen (BUN) levels

The AST, ALT and BUN levels in the serum were determined using enzyme-linked immunosorbent assay (ELISA) kits (Shanghai Institute of Biological Products Co., Ltd., Shanghai, China).

### Splenocyte proliferation assay

Splenocytes were obtained by gentle disruption of the spleen of the Balb/c female mice and filtration via a 40-μm Nylon cell strainer (Falcon, NJ, USA). The erythrocytes were lysed with 0.38% NH_4_Cl-Tris buffer (pH 7.4), while the remaining cells were resuspended in RPMI-1640 with 10 mM Hepes, 10% fetal bovine serum, 100 mg/l streptomycin and 100 IU/ml penicillin.

The splenocytes (1×10^6^ cells/ml) were treated with mitogens, including lipopolysaccharide (LPS) and concanavalin A (Con A) (Grand Island Biological Co., Grand Island, NY, USA), at 10 μg/ml, and co-cultured with the test samples in 24-well plates for 24, 48 and 72 h at 37°C in a humidified atmosphere of 5% CO_2_. The proliferation of the splenocytes was measured using a 3-(4,5-dimethyl-2-thiazolyl)-2,5-diphenyltetrazolium bromide assay (12).

### ELISA analysis of serum inflammation-related cytokines

For the serum cytokine assay, blood from the inferior vena cava was collected into a tube and centrifuged at 1,800 × g for 10 min at 4°C. The serum was aspirated and assayed as follows. The serum concentrations of the inflammatory-related cytokines, tumor necrosis factor (TNF)-α and interleukin (IL)-1β (Biolegend, San Diego, CA, USA), were measured by ELISA, according to the manufacturer’s instructions (Biolegend, CA, USA). Briefly, subsequent to adding the biotinylated antibody reagent to the 96-well plates, the supernatants of the homogenized serum were incubated at 37°C in CO_2_ for 2 h. Subsequent to being washed with PBS, streptavidin-horseradish peroxidase solution was added and the plate was incubated for 30 min at room temperature. The absorbance was measured at 450 nm using a microplate reader (model 680, Bio-Rad, Hercules, CA, USA) (13).

### Statistical analysis

Analysis of variance (ANOVA) was performed and the results are presented as the mean ± standard deviation. The differences between the mean values of the individual groups were assessed using one-way ANOVA and Duncan’s multiple range tests. P<0.05 was considered to indicate a statistically significant difference. The SAS v9.1 statistical software package (SAS Institute Inc., Cary, NC, USA) was used to perform all the statistical analyses.

## Results

### Tumor growth inhibitory effects of Juemingzi

The antitumor activity of Juemingzi was tested against the sarcoma 180 tumor-bearing mice. The average tumor weight of the sarcoma 180 cell-bearing mice receiving a normal diet (control) was 4.8±0.4 g, whereas that of the mice that were treated with 50 and 100 mg/kg b.w. Juemingzi was 4.1±0.5 and 3.3±0.5 g, respectively and that of the mice that were treated with 200 mg/kg b.w. Juemingzi was 2.9±0.4 g. Juemingzi extract (50, 100 and 200 mg/kg b.w.)-treated mice demonstrated tumor growth inhibitory rates of 14.6, 31.3 and 39.6%, respectively. According to the results, a good antitumor effect was observed in the tumors that were treated with high concentrations of Juemingzi (Table I).

### Effect of Juemingzi on serum AST, ALT and BUN levels

**Figure 1:**
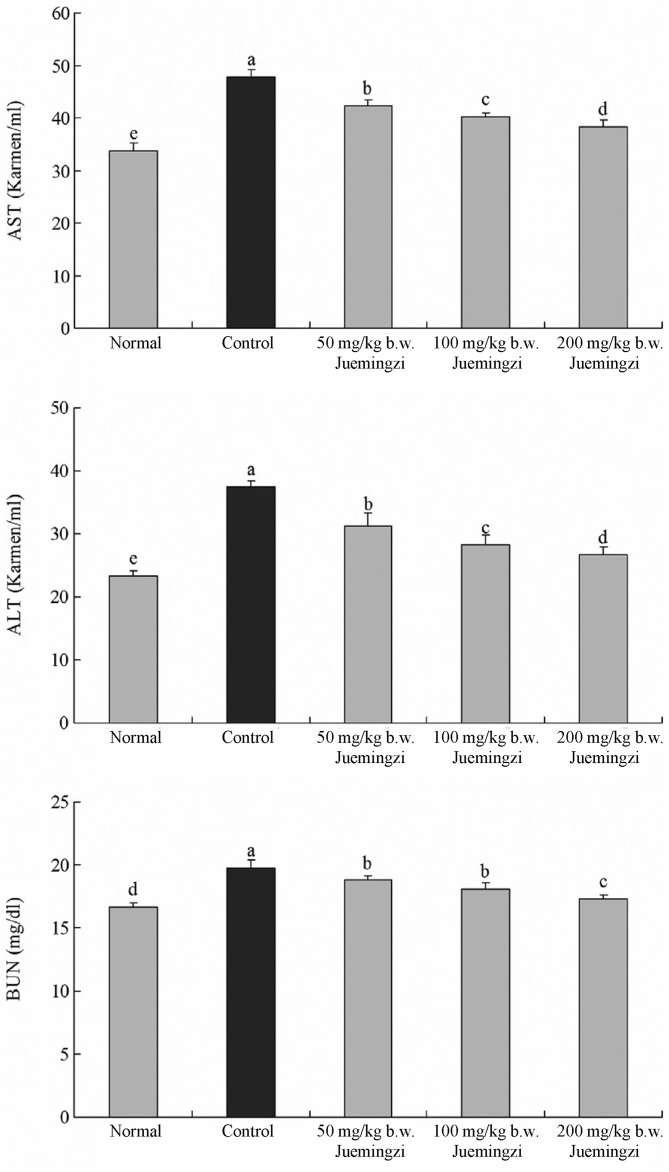
Serum AST, ALT and BUN levels of Juemingzi-treated sarcoma 180 tumor-bearing mice. ^a–e^Mean values with different letters over the bars are significantly different (P<0.05), according to Duncan’s multiple range test. AST, aspartate aminotransferase; ALT, alanine transaminase; BUN, blood urea nitrogen.

The AST levels in the normal mice were 33.8±1.4 Karmen U/ml. However, that of the sarcoma 180 control mice was significantly increased to 47.9±1.2 Karmen U/ml. The levels of AST in the mice that were treated with 50, 100 and 200 mg/kg b.w. Juemingzi were 42.2±1.2, 40.1±0.8 and 38.3±1.4 Karmen U/ml, respectively (Fig. 1).

The ALT levels in the normal group were 23.3±0.8 Karmen U/ml, whereas those in the control group were 37.4±1.0 Karmen U/ml, reflecting a marked increase. The ALT levels in the 50-, 100- and 200-mg/kg b.w. Juemingzi groups decreased to 31.2±2.2, 28.3±1.6 and 26.6±1.4 Karmen U/ml, respectively (Fig. 1).

The levels of BUN in the 50-, 100- and 200-mg/kg b.w. Juemingzi groups were 18.8±0.3, 18.1±0.5 and 17.3±0.3 mg/dl, respectively, which were marginally lower than those of the control group (19.8±0.6 mg/dl). However, the BUN levels in the 200 mg/kg b.w. normal group were 16.7±0.3 mg/dl (Fig. 1). The AST, ALT and BUN levels in the Juemingzi groups were lower than those in the control group and the mice from the 200-mg/kg b.w. Juemingzi group demonstrated levels that were similar to those of the normal group.

### Effects of Juemingzi on lymphocyte proliferation

**Figure 2:**
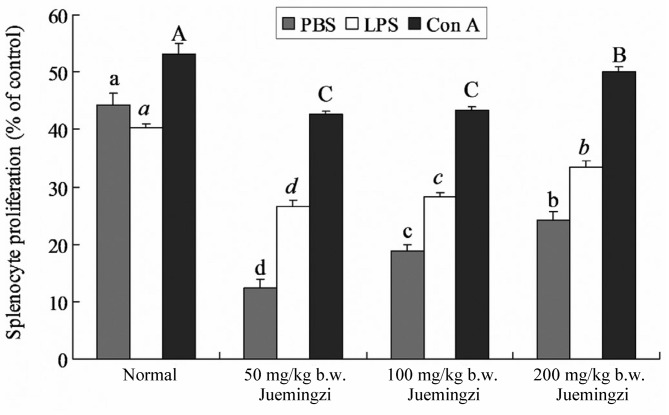
Effect of Juemingzi on splenocyte cell proliferations with PBS, 2 μg/ml LPS, Con A for 24 h. ^a–d,^*^a–d^*^,A–C^Mean values with different letters over the bars are significantly different (P<0.05), according to Duncan’s multiple range test. PBS, phosphate-buffered saline; LPS, lipopolysaccharide; Con A, concanavalin A.

The lymphocytes of the sarcoma 180 tumor-bearing mice in each group were irritated by LPS or Con A. The irritated lymphocytes were compared with the non-irritated PBS lymphocytes. As shown in Fig. 2, after 24 h, the proliferation rate of the Con A-treated lymphocytes was greater than that of the LPS-treated lymphocytes. The Con A-treated lymphocytes in the 200-mg/kg b.w. Juemingzi group (50.1%) demonstrated a marginally lower lymphocyte proliferation rate than that of the normal group (53.2%). The lymphocyte proliferation rate in the 50- and 100-mg/kg b.w. Juemingzi groups was 42.7 and 43.4%, respectively. By these results, the Con A-treated lymphocyte proliferation rate was higher in the group that was treated with a high concentration of Juemingzi compared with those that were treated with a lower concentration.

### Effect of Juemingzi on serum TNF-α and IL-1β levels

**Figure 3:**
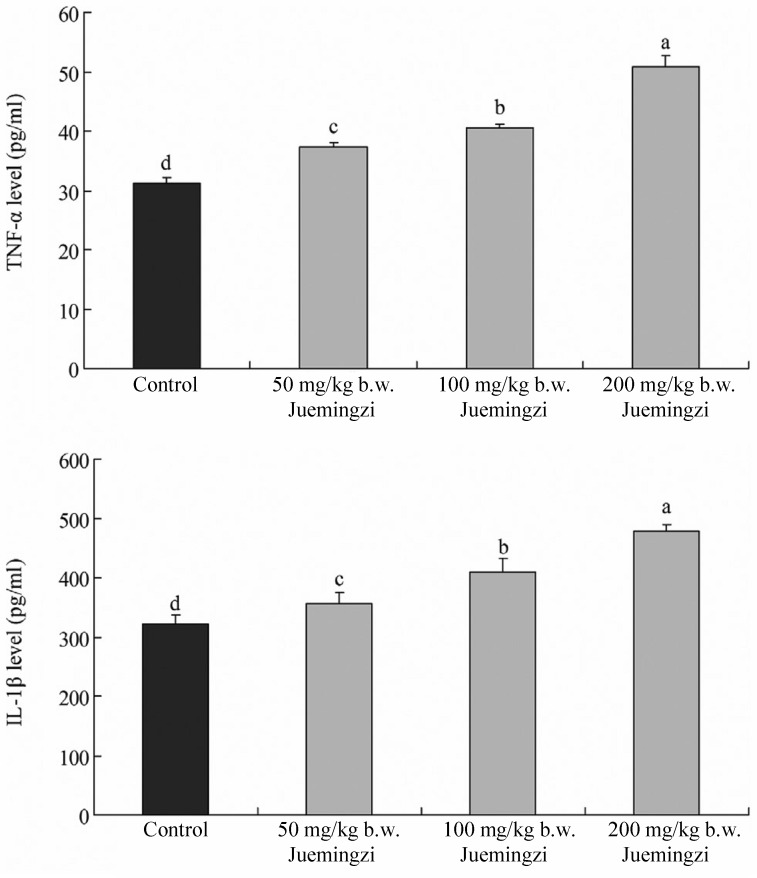
Serum TNF-α and IL-1β levels of Juemingzi-treated sarcoma 180 tumor-injected mice. ^a–e^Mean values with different letters over the bars are significantly different (P<0.05), according to Duncan’s multiple range test. TNF, tumor nercrosis factor; IL, interleukin.

The serum TNF-α and IL-1β levels of the mice in the Juemingzi-treated groups were significantly higher than those in the control group (Fig. 3). The increases in the TNF-α and IL-1β levels in the 200 mg/kg b.w. Juemingzi-treated mice were 50.8±2.0 and 478.3±10.5 pg/ml, respectively, compared with those of the control group (31.2±1.0 and 322.7±14.3 pg/ml). The TNF-α levels in the mice that were treated with 50 and 100 mg/kg b.w. Juemingzi were 37.3±0.8 and 40.5±0.7, respectively and the IL-1β levels were 355.9±19.2 and 408.6±24.1, respectively.

## Discussion

Although Juemingzi has been used in medicine, the scientific data concerning its effects are limited. Juemingzi has previously been demonstrated to have various therapeutic effects on numerous pathological conditions, including inflammation, aging and cancer (14).

Cell injury can release endogenous damage-associated molecular patterns that activate innate immunity. Cell injury can also increase the risk of cancer (15). AST and ALT are enzymes that are located in cells that leak out into the general circulation when cells are injured. AST is located in a number of body tissues, including the heart, muscle, kidney, brain and lung tissues (16). A decreased BUN:creatinine ratio indicates malnutrition and syndrome of inappropriate antidiuretic hormone secretion, which is occasionally observed with lung diseases, cancer and diseases of the central nervous system (17).

Lymphocytes are a type of white blood cell in the vertebrate immune system. The three major types of lymphocyte are T cells, B cells and natural killer (NK) cells (18). NK cells are a part of the innate immune system and play a major role in defending the host from tumors and virally infected cells. NK cells distinguish infected cells and tumors from normal and uninfected cells by recognizing changes in the surface MHC class I molecule. NK cells are activated in response to a family of cytokines called interferons. Activated NK cells release cytotoxic (cell-killing) granules, which then destroy the altered cells (19).

TNF-α and Il-1β are significant regulators of host defense against tumor cells (20). The observed increase in the production of these cytokines may suggest an enhanced ability of the host to combat the growth of tumors. Macrophages are activated by β-glucans and other cell mediators to kill tumor cells by the production of TNF-α and ILs. The bioactivities of polysaccharides and polysaccharide-protein complexes are dependent on the binding of the surface receptor of immune cells. These receptors, known as pattern recognition molecules, are able to recognize foreign ligands during the initial phases of the immune response (21). At high levels of IL-1β, cancer cells that receive genotoxic insults engage the apoptotic pathways. Cotreatment with an inhibitor of IL-1β and TNF-α synthesis has been demonstrated to prevent carcinogen-induced lesions (22). Oxidation is highly reactive and has the potential to cause damage to cells, including damage that may lead to cancer. Juemingzi demonstrated a preventive effect against atherosclerosis by inhibiting LDL oxidation (23). Additionally, Juemingzi was found have *in vitro* anticancer effects in cancer cells (24). The present study provided further data indicating the *in vivo* anticancer effects of Juemingzi.

The results from the present study have demonstrated that Juemingzi is able to decrease sarcoma 180 tumors. Juemingzi exhibited strong activity, as observed by the tumor weight count, serum levels assay and lymphocyte proliferation rate. An increased concentration of Juemingzi is significant for augmenting these antitumor effects on sarcoma 180-treated tumors. The active compounds obtained from Juemingzi (*Cassia tora* L.) need to be identified and evaluated in future studies.

## Figures and Tables

**Table I:** Anti-tumor activities of 50, 100 and 200 mg/kg b.w. Juemingzi-treated sarcoma 180 tumor-injected mice.

Group	Tumor weight (g)	Inhibitory rate (%)
Control	4.8±0.4^a^	
Juemingzi		
50 mg/kg b.w.	4.1±0.5^b^	14.6
100 mg/kg b.w.	3.3±0.5^c^	31.3
200 mg/kg b.w.	2.9±0.4^d^	39.6

a–d Mean values with different letters in the same column are significantly different (P<0.05), according to Duncan’s multiple-range test.
